# Allergenic Characterization of New Mutant Forms of Pru p 3 as New Immunotherapy Vaccines

**DOI:** 10.1155/2013/385615

**Published:** 2013-11-14

**Authors:** C. Gómez-Casado, M. Garrido-Arandia, P. Gamboa, N. Blanca-López, G. Canto, J. Varela, J. Cuesta-Herranz, L. F. Pacios, A. Díaz-Perales, L. Tordesillas

**Affiliations:** ^1^Centro de Biotecnología y Genómica de Plantas (UPM-INIA), Campus de Montegancedo, Pozuelo de Alarcón, 28223 Madrid, Spain; ^2^Servicio de Alergia, Hospital de Basurto, Bilbao, Spain; ^3^Servicio de Alergia, Hospital Infanta Leonor, Madrid, Spain; ^4^Servicio de Quimica de Proteinas, Centro de Investigaciones Biologicas, CSIC, Madrid, Spain; ^5^IIS-Servicio de Alergia, Fundación Jiménez Díaz, Madrid, Spain; ^6^Departamento de Biotecnología, E.T.S. Ingenieros de Montes, UPM, Madrid, Spain

## Abstract

Nowadays, treatment of food allergy only considered the avoidance of the specific food. However, the possibility of cross-reactivity makes this practice not very effective. Immunotherapy may exhibit as a good alternative to food allergy treatment. The use of hypoallergenic molecules with reduced IgE binding capacity but with ability to stimulate the immune system is a promising tool which could be developed for immunotherapy. In this study, three mutants of Pru p 3, the principal allergen of peach, were produced based on the described mimotope and T cell epitopes, by changing the specific residues to alanine, named as *Pru p 3.01, Pru p 3.02*, and *Pru p 3.03*. *Pru p 3.01* showed very similar allergenic activity as the wild type by *in vitro* assays. However, *Pru p 3.02* and *Pru p 3.03* presented reduced IgE binding with respect to the native form, by *in vitro*, *ex vivo,* and in vivo assays. In addition, *Pru p 3.03* had affected the IgG4 binding capacity and presented a random circular dichroism, which was reflected in the nonrecognition by specific antibodies anti-Pru p 3. Nevertheless, both *Pru p 3.02 *and *Pru p 3.03* maintained the binding to IgG1 and their ability to activate T lymphocytes. Thus, *Pru p 3.02* and *Pru p 3.03* could be good candidates for potential immunotherapy in peach-allergic patients.

## 1. Introduction

IgE-mediated allergy is a hypersensitivity disease suffering from more than 25% of the population in industrialized countries. Currently, specific immunotherapy is the only allergen-specific approach for its treatment and for preventing its progression to severe manifestations [1, 2]. The administration of increasing doses of allergen extracts to patients is the method most commonly applied. However, the use of crude extracts has several disadvantages. It could induce severe anaphylactic side reactions [3] or lead to sensitization towards new allergens present in the mixture [4, 5]. Different strategies have been designed to try to overcome these negative effects [6], as the use of allergen-derived B cell peptides [7, 8], allergen-derived T cell epitope containing peptides [9, 10] or vaccination with allergen-encoding DNA [11, 12]. In food allergy, immunotherapy is not commonly used due to the negative side effects, although several studies have been performed [13, 14].

The use of hypoallergenic mutants would be a good strategy to avoid the nondesired side effects of immunotherapy. Hypoallergenic mutants have been developed for several pollens and foods allergens [15–18], and their utility for immunotherapy has been studied [19, 20]. These mutants have altered their capacity to bind IgE, but they still preserve the capacity to stimulate the immune system, inducing the proliferation of T lymphocytes and the production of specific blocking IgG antibodies which compete with IgE. Different strategies have been designed to develop these mutants [21–23], such as destruction of conformation, site-directed mutagenesis, or oligomerization.

Peach allergy is the most prevalent plant food allergy in the Mediterranean area, and its major allergen, the LTP, Pru p 3, is the main plant food allergen in this region [24, 25]. The current management of peach allergy is to avoid its ingestion, both fresh and processed forms, due to the fact that it could induce potentially severe reactions [26, 27]. B and T cell epitopes have been characterized in Pru p 3 [28–30], being conserved in other LTPs [31].

In the case of the LTPs, some hypoallergenic forms have been developed by altering the structure of the protein, such as in Par j 1, pellitory pollen LTP, and Pru p 3 [16, 17]. The hypoallergenic *Par j 1* was developed with the T cell epitope and IgG responses not affected [16]. However, the Pru p 3 mutant lost the capacity to bind specific IgG antibodies in mice, which could be a problem in the process of a successful immunotherapy [17, 32, 33]. Recently, a hybrid molecule has been characterized as hypoallergenic mutant, and its application in immunotherapy is possible [34]. The two allergenic LTPs from pellitory pollen, Par j 1 and Par j 2, were merged and produced as recombinant proteins. The hybrid showed a decrease in its allergenic capacity [34].

Based on these studies and the characterized Pru p 3 epitopes, we produced three mutant forms of Pru p 3*, Pru p 3.01, Pru p 3.02, and Pru p 3.03*, as recombinant proteins, using site-directed mutagenesis of residues included in its recently identified epitope [29, 31]. The new LTP forms *Pru p 3.02* and *Pru p 3.03 *showed a decrease in their IgE binding capacity with respect to the wild type, with *in vitro*, *ex vivo* and *in vivo* evidence. However, they conserved their IgG epitopes and retained their capacity to stimulate T cells, inducing similar cytokine profiles to the wild type allergen.

## 2. Methods

### 2.1. Patients and Sera

Sera from 10 patients with allergy to peach selected at the Allergy Service of the Fundación Jiménez Díaz (Madrid), Hospital de Basurto (Bilbao), and Hospital Infanta Leonor (Madrid) were used (age: 16–46; sex: 55% female, 45% male). All patients had a convincing clinical history of immediate allergic reactions after peach ingestion (urticaria/angioedema or anaphylactic symptoms); a positive response in the skin prick test (SPT) using a commercial peach peel extract (ALK-Abello, Madrid, Spain); and a positive response to peach by open oral challenge, except patients that had suffered from anaphylaxis [35, 36].

Written informed consent was obtained from all patients and the study was approved by the ethics committees of the corresponding hospitals (Fundación Jiménez Díaz, Madrid, spain; Hospital de Basurto, Bilbao, Spain; Hospital Infanta Leonor, Madrid, Spain).

### 2.2. Production of Wild Type and Mutant Forms of Pru p 3

Site-directed mutagenesis to generate Pru p 3 mutants was performed with appropriate PCR amplification primers and, as a template, the previously obtained cDNA encoding Pru p 3 [37]. All substitutions were made to alanine. The production of recombinant proteins was performed in *Pichia pastoris* yeast following the manufacturer's recommendations (Invitrogen Corporation, De Schelp, The Netherlands). The recombinant proteins were purified from induced supernatants by two chromatographic steps: gel filtration on a Superdex HR 75 16/26 column (Pharmacia Biotech, Piscataway, NJ, USA) followed by RP-HPLC on a Nucleosil 300-C4 column (7 × 250 mm; particle size 5 *μ*g; Tecknokroma, Barcelona, Spain).

Purified proteins were quantified by means of the commercial bicinchoninic acid test (Pierce, Cheshire, UK), and purity was measured by SDS-PAGE, N-terminal amino acid sequencing with an Applied Biosystems 477A gas-phase sequencer (Applied Biosystems, Foster City, CA, USA), mass spectrophotometric analysis with a Biflex III Spectrometer (Bruker-Franzen Analytik, Bremen, Germany), and fingerprinting after tryptic digestion, using standard methods.

Circular dichroism (CD) measurements were performed on a Jasco Model J-720 spectropolarimeter (Japan Spectroscopic Co., Tokyo, Japan), using a quartz cell with a 1 mm light path length, thermostatically controlled with a Jasco Model 423S Peltier-type temperature controller, and a thermostated cell holder, interfaced with a thermostatic bath. The far-UV spectra were recorded at 20°C from 190 to 260 nm, as an average of five scans, after being corrected by sustration of a buffer blank. The protein concentration was in the range 0.2–0.25 mg/mL. Mean residue mass ellipticities were expressed in terms of (−) (degree cm^2^ dmol^−1^).

To compare the activity of wild type with the mutant forms, the concentration of the proteins was calibrated by amino acid analysis following the standard ion-exchange chromatographic method in a Pharmacia Biochrom 20 analyzer.

For cell culture, the absence of LPS in the sample was checked by anti-LPS antibodies (rabbit anti-*Escherichia coli* LPS; AbD Serotec, Kidlington, UK) and using THP1-XBlue cells (Invivogen, Toulouse, France).

### 2.3. Specific IgE and IgGs ELISA Assays

Specific IgE, IgG1, and IgG4 were determined by direct ELISA assay as previously reported [38, 39], using sera from patients, goat anti-human IgE peroxidase (Biosource, Camarillo, CA, USA), and mouse anti-human-IgG1 or -IgG4 antibodies (Invitrogen) with the corresponding secondary antibodies. Blocking solution (Sigma, St. Louis, MO, USA) without solid phase was used as negative control, and O.D. values greater than mean [OD] + 3x SD to the negative control were considered positive. The sera dilution was determined by titration curves.

ELISA-inhibition assays were carried out incubating single sera or pool (3 h at 25°C) with the corresponding inhibitor before adding the solution to the solid phase. The appropriate concentration of the inhibitor was selected by titration curves.

### 2.4. Basophil Activation Test (BAT)

Wild type Pru p 3 and the mutant forms, *Pru p 3.01, Pru p 3.02, and Pru p 3.03*, were tested by performing the BAT with whole blood samples from 4 peach-allergic donors (Table 1), as previously described [40, 41]. Blood samples were obtained from 4 patients at the Servicio de Alergia, Fundación Jiménez Díaz (Madrid) and Hospital Infanta Leonor (Madrid). The criteria for the selection were the same as described above.

Following separation of blood cells, 50 *μ*L of the patient's cell suspension was incubated with 50 *μ*L of two final concentrations of the tested samples: 10 and 1 *μ*g/mL for Pru p 3, *Pru p 3.01, Pru p 3.02,* and* Pru p 3.03*. As a positive control, a monoclonal anti-IgE receptor antibody (Bethyl Laboratories, Montgomery, TX, USA) was used at a final concentration of 1 *μ*g/mL. The test was considered positive when the % of positive response was >15% and the stimulation index (SI, calculated as the percentage of activated basophils with allergen to the percentage of activated basophils at baseline conditions) was ≥2. The dilution corresponding to 1 *μ*g/mL was selected as the most appropriate for these samples.

### 2.5. Proliferation Assays with T Cell Lines from Peach-Allergic Patients

PBMCs were freshly isolated from whole blood subjected to density gradient centrifugation on Lymphoprep (Axis-Shield, Oslo, Norway), as previously described [30]. Cultures were established in 24-well plates (Costar, NY, USA) at 2 × 10^6^ cells per well and treated with Pru p 3 (10 *μ*g/mL) in RPMI media (Invitrogen), supplemented with 10% (v/v) of fetal calf serum (Invitrogen), 0.02 mM mercaptoethanol, 2 mM glutamine, and 10 mM HEPES at 37°C, in a 5% CO_2_ humidified atmosphere. After 5 days, a half-volume medium was removed and suboptimal doses of rIL2 (10 ng/mL; Invitrogen) were added. Cultures were continued for an additional 7 days. On the 12th day, fresh media supplemented with rIL2 was added in the presence of 10^5^ autologous-mitomycin-treated PBMCs. Finally (on the 19th day of culture), specific TCLs (5 × 10^4^ cells per well) and their mitomycin-treated PBMCs (10^5^ cells per well) were seeded in 96 wells. The allergens, peptides, or mutants were added to the TCL culture in triplicate wells for 48 h. Within the last 16 h, [^3^H]-thymidine (0.5 *μ*Ci/well) was added, and the incorporated radioactivity was measured by scintillation counting. Phytohemagglutinin-L from *Phaseolus vulgaris* (PHA, 1 *μ*g/mL; Roche, Mannheim, Germany) was used as a positive control. The stimulation index (SI) was calculated as the ratio between counts of antigen-stimulated cultures and counts without any activator. A SI ≥ 2 was considered a positive value.

### 2.6. HLA Class II Blocking Assays

To demonstrate the specificity of the proliferation in TCLs experiments, human leukocyte antigen (HLA) class II inhibition assays were performed using specific antibodies, as previously described [42]. Specific TCLs (5 × 10^4^ cells per well) and their mitomycin-treated PBMCs (10^5^ cells per well) were seeded in 96 wells (see above). They were incubated with 0.5 and 5 *μ*g/mL of anti-HLA-DP/DQ/DR (AbSerotec, Dusseldorf, Germany) or isotype control antibodies (Alpha Diagnostic International Inc., San Antonio, TX, USA) for 2 h at 37°C before addition of the corresponding allergen or its mutants and testing of the proliferative response.

### 2.7. Determination of IL4, IFN*γ*, and IL10 Levels in TCL Cultures

Cytokine levels were quantified using 50 *μ*L of TCL supernatants, by means of matched antibody pairs according to the manufacturer's instructions (sensitive limits: IL4, 0.6 pg/mL; IFN*γ*, 2 pg/mL; IL10, 1.2 pg/mL; ImmunoTools, Friesoythe, Germany). Cultures containing TCLs with mitomycin-treated PBMCs alone served as negative controls. IFN*γ*/IL4 ratios over 10, between 2 and 10, and less than 2 were, respectively, considered as Th1, Th0, and Th2-responses.

### 2.8. Phenotype Analysis of TCLs Using Real-Time PCR

mRNA from the TCLs was isolated according to the Quiagen-RNeasy protocol (Quiagen, Valencia, CA, USA) and stored at −80°C. RT-PCR was performed as previously described [37, 43]. cDNA was amplified using the Power SYBR Green PCR Master Mix (Applied Biosystems) according to the manufacturer's recommendations and run on aa Applied Biosystems 7300 real-time detection system (Applied Biosystem), using previously described primers (GATA-3, F: 5′-GCGGGCTCTATCACAAAAT GA-3′, R: 5′-GCTCTCCTGGCTGCAGACAGC-3′; Fox p 3, F: 5′-GAAACAGCACATTCCCAGAGTTC-3′, R: 5′-ATGGCCCAGCGGATGAG-3′; T-bet, F: 5′-GATGCGCCAGGAAGTTTCAT-3′, R: 5′-GCACAATCATCTGGGTCACATT-3′; ROR*γ*, F: 5′-AAATCTGTGGGGACAAGTCG-3′, R: 5′-TGAGGGTATCTGCTCCTTGG-3′; EF-1, F: 5′-CTGAACCATCCAGGCCAAAT-3′, R: 5′-GCCGTGTGGCAATCCAAT-3′; [44, 45]. Real-time PCR conditions were as follows: 10 min at 95°C, 15 s at 95°C, and 60 s at 60°C (40 cycles). The amount of GATA-3, Fox p 3, T-bet, and ROR*γτ* mRNA expression was normalized with endogenous control EF-1, and the relative quantification was performed using the comparative threshold cycle method (2^−ΔΔCt^), as described by Livak and Schmittgen [46]. The changes in gene expression were calculated with respect to the untreated cells. All amplifications were carried out in duplicate.

### 2.9. Statistical Analysis

Statistical analysis was performed using SPSS 17.0 and Statgraphics Centurion XVI. Wild type and mutants were compared using the Wilcoxon paired samples test, in the Ig binding ELISA assays, proliferative responses of T cell lines, cytokine levels, and gene expression. Values of *P* < 0.05 were considered to be significant.

## 3. Results

### 3.1. The Mutant Forms Showed a Reduced IgE Binding Capacity

**Figure 1:**
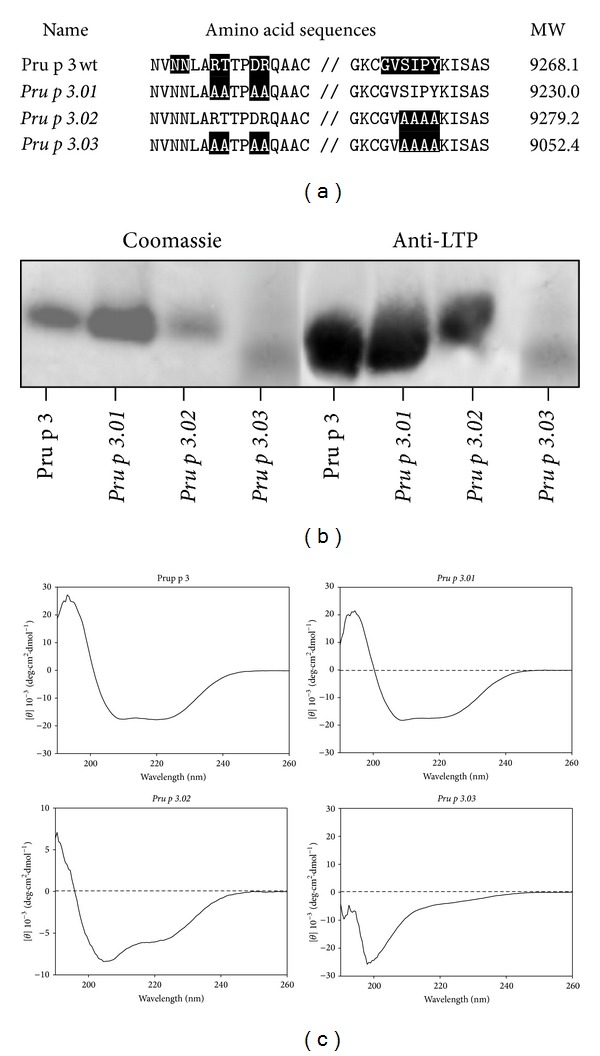
(a) Alignment of amino acid sequences of the wild type (Pru p 3) and mutant forms (*Pru p 3.01, Pru p 3.02*,and* Pru p 3.03*). The residues shaded in black correspond to the IgE epitope in Pru p 3 or the modified residues in *Pru p 3.01, Pru p 3.02*, and *Pru p 3.03*. The molecular weight (MW) obtained by mass spectrometry is indicated. (b) The recombinant proteins (3 *μ*g) were separated by SDS-PAGE and stained with Coomassie Blue (Coomassie) or immunodetected with anti-Pru p 3 antibody (anti-LTP). (c) Far-UV (190–260 nm) CD spectrum of purified proteins.

 Pru p 3 residues involved in B epitopes [29, 31] were modified by site-directed mutagenesis, producing three mutants as recombinant proteins (Figure 1(a)). The three mutated forms corresponded to a single peak by both HPLC and mass spectrometry and yielded a unique amino terminal sequence. Similarly, *Pru p 3.01 *and* Pru p 3.02* could be detected as a single band by both Coomassie staining and polyclonal antibodies to the wild type protein (Figure 1(b)). However, *Pru p 3.03* was observed as multiple bands staining with Coomassie, and it was slightly detected by Pru p 3-specific antibody. Evidence for the folding of the mutants was obtained by CD analysis in the far-UV range (Figure 1(c)). Application of the convex-constraint analysis method to the spectrum resulted in a great increase of the random coil in the *Pru p 3.03* form comparing to the wild type. By contrast, the *Pru p 3.01* and *Pru 3.02* forms showed an overlapped spectrum with the native form.

**Figure 2:**
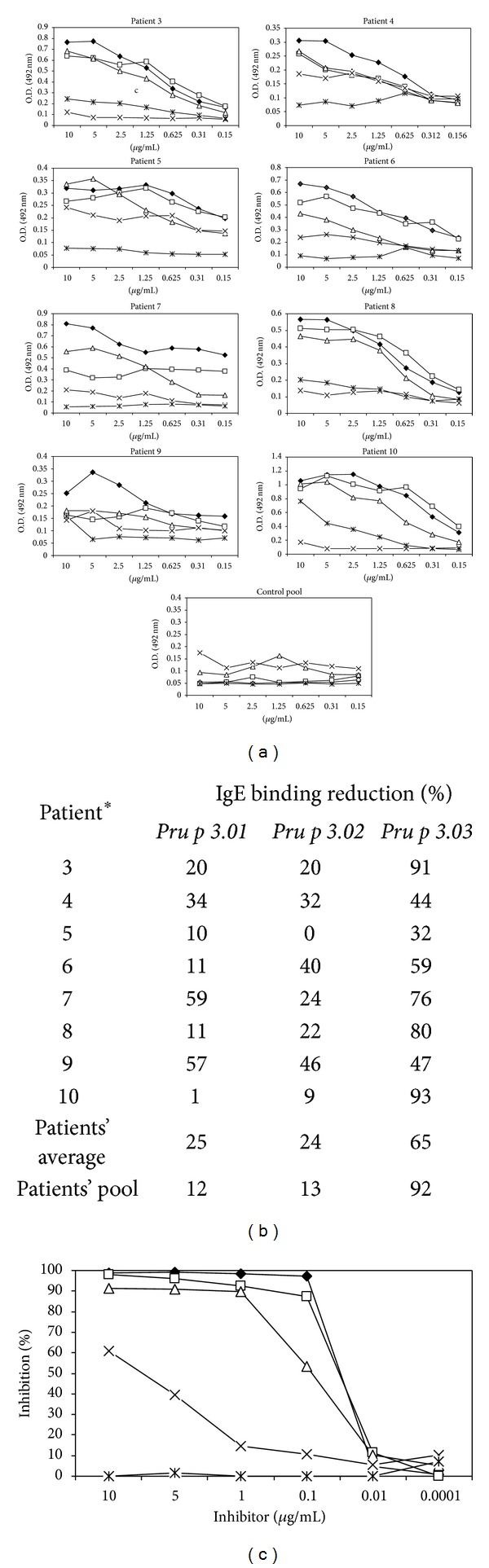
(a) Specific IgE to Pru p 3 forms (wild type, *Pru p 3.01, Pru p 3.02,* and *Pru p 3.03*), using sera from peach-allergic patients (single or pool; 1 : 2 dilution). Blocking solution without solid phase was used as a negative control, and optical density (OD) values > 0.189 units (*n* = 30; mean + 3x SD = 0.108 + 3 × 0.026 OD units) were considered positive. All tests were performed in triplicate. (b) Reduction of IgE binding capacity (%) of the mutants in comparison with wild type was calculated, taking as 100% the value obtained for Pru p 3 (5 *μ*g/mL). *The number of the patients corresponds to that in Table 1. (c) The inhibition percentage of the IgE binding capacity of wild type form is represented. Plates were covered with Pru p 3 (3 *μ*g/mL), and *Pru p 3.01, Pru p 3.0*, and* Pru p 3.03 *were used as inhibitors. All tests were performed in triplicate.

 Referring to allergenic activity, *Pru p 3.03* showed a significant reduction in the IgE binding capacity, compared to the wild type. The recognition of this mutant form by peach-allergic patients was low, ranging from 32 to 93% of reduction in IgE binding capacity compared to the wild type (Figures 2(a) and 2(b)), this reduction being statistically significative (*P* = 0.0001). These data suggested that B epitopes in *Pru p 3.03* were altered. The decrease of *Pru p 3.03*-IgE binding capacity was confirmed by inhibition ELISA, using a serum pool from peach-allergic patients, and Pru p 3 wild type as solid phase (Figure 2(c)). *Pru p 3.03* rendered low inhibition values of the IgE binding of the form, reaching only 60% of inhibition.

In the case of the other two mutants, *Pru p 3.01* and *Pru p 3.02*, the reduction in the IgE binding was not as dramatic as in the case of *Pru p 3.03*, with only a 12-13% of reduction for the serum pool (Figures 2(a) and 2(b)). By contrast, the reduction values of single sera ranged from 1 to 59% for *Pru p 3.01* and from 0 to 46% for *Pru p 3.02, *being statistically significative in the case of the latter (*P* = 0.0023). Both forms, *Pru p 3.01* and *Pru p 3.02, *almost completely inhibited the IgE binding, being a little lower in the case of *Pru p 3.02* (Figure 2(c)).

### 3.2. The Three Pru p 3 Mutants Retained Their Ability to Bind IgG Blocking Antibodies

**Figure 3:**
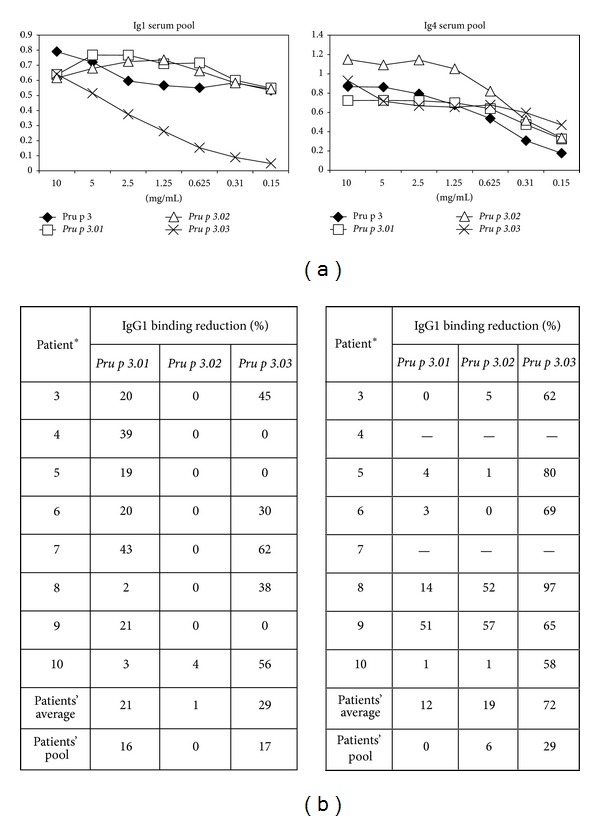
(a) Specific IgG1 and IgG4 to Pru p 3 forms (wild type, *Pru p 3.01, Pru p 3.02*, and *Pru p 3.03*), using serum pool from peach-allergic patients (1 : 10 dilution). Blocking solution without solid phase was used as a negative control, and optical density (OD) values > 0.086 units (*n* = 20; mean + 3x SD = 0.05 + 3 × 0.012 OD units) were considered positive. All tests were performed in triplicate. (b) Reduction of IgG1 and IgG4 binding capacity (%) of the mutants in comparison with Pru p 3 was calculated by taking the value of Pru p 3 (5 *μ*g/mL) to represent 100% in each serum sample. *The number of the patients corresponds to that in Table 1. −, negative value, represents those patients with negative IgG4 for Pru p 3.

To test the capacity to bind IgG blocking antibodies, direct ELISA assays were also performed against IgG1 and IgG4 (Figure 3). The three mutant forms could bind IgG1, without being statistically significant in the case of *Pru p 3.01* and *Pru p 3.03* (*P* = 0.381 and 0.413, resp.; Figure 3), indicating that the IgG1 epitopes were mostly conserved. Curiously, *Pru p 3.02* rendered differences with respect to the wild type (*P* = 0.0083) because higher values were obtained with the mutant (Figure 3).

On the other hand, several patients showed low levels of specific IgG4 against the native form of Pru p 3 (Figure 3). In the case of patients with positive response, the IgG4 binding was similar in *Pru p 3.01* and *Pru p 3.02* (*P* = 0.889 and 0.129, resp.), indicating that these mutants have not affected these epitopes (Figure 3). Nonetheless, the binding of *Pru p 3.03* was decreased (*P* = 0.0001), with values ranging from 0 to 97% of reduction, indicating that the IgG4 epitopes were affected, at least in part.

### 3.3. Reduced *Ex Vivo* Allergic Response Induced by the Mutants

The reduction in the IgE binding observed *in vitro* was confirmed by *ex vivo* assays, because most patients did not recognize the Pru p 3 mutant forms (Table 1). The mutants did not activate basophils from patients with peach allergy in most cases (Table 1: 2/4 positive cases *Pru p 3.01*; 1/4 positive cases in the case of *Pru p 3.02* and *Pru p 3.03*).

### 3.4. The T Cell Activation Capacity Was Maintained by the Pru p 3 Mutants

**Figure 4:**
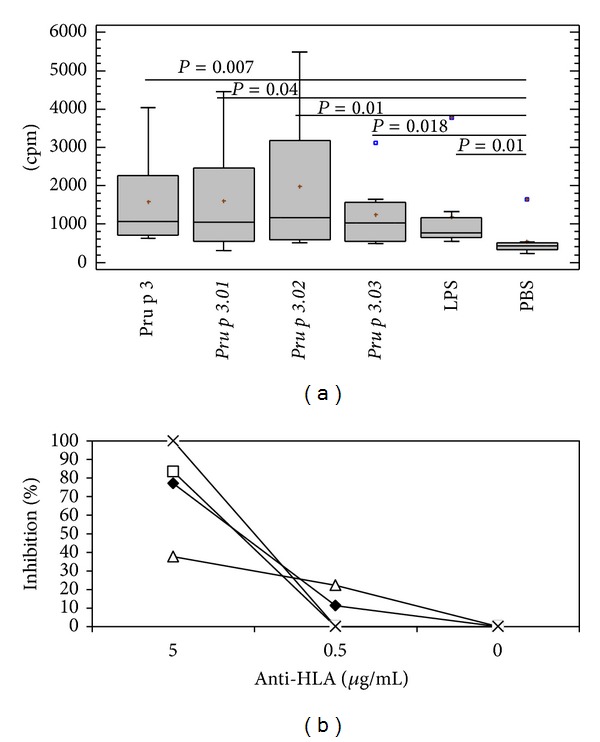
(a) Proliferative response of Pru p 3 T cell lines from peach-allergic patients to wild type, mutants (*Pru p 3.01, Pru p 3.02*, and *Pru p 3.03*), LPS, and negative control (PBS), as a measure of incorporated radioactivity. Medians (—) and range (bars) were calculated from *n* = 24 values for each protein. Significant differences between the antigens and untreated culture (PBS) are indicated. (b) HLA-II restriction specificity of T cell recognition of wild type and the mutant forms. The inhibition percentage of the proliferation of specific TCLs to each protein in the presence of anti-HLA antibodies is represented for patient 2, as an example.

The proliferative response of Pru p 3 specific T-cell lines was measured after culture in presence of the wild type and mutant forms (Figure 4(a)). The stimulation index for the positive control (PHA) ranged from 2.6 to 59.8 (data not shown). The mutant forms induced a slightly lower response compared to the wild type, although no statistical differences were observed (*P* = 0.363, *P* = 0.958, and *P* = 0.494, for *Pru p 3.01*, *Pru p 3.02*, and *Pru p 3.03*, resp.). So, these results suggest that the mutations did not affect the T cell epitopes. This proliferation was prevented by incubation with anti-HLA antibody in a dose-dependent manner (Figure 4(b)). Isotype control antibody had no effect (data not shown).

**Figure 5:**
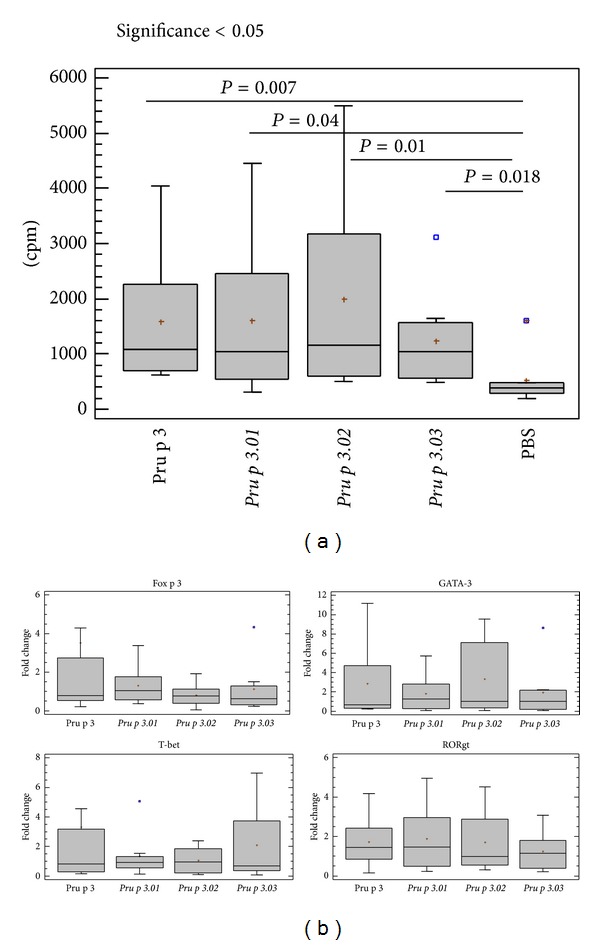
(a) The ratio between IFN*γ* and IL4 for T cell line supernatants stimulated with Pru p 3 and its mutant forms is represented (*n* = 6). Statistical analysis was performed by a paired-samples Wilcoxon test (*P* < 0.05). Medians (—) and ranges (bars) are shown. (b) GATA-3, Fox p 3, T-bet, and ROR*γτ* relative gene expression levels for T cell lines from peach-allergic patients (*n* = 6). The results are expressed in fold change comparing nonstimulated with stimulated conditions, and statistical significance is indicated (*P* < 0.05) after performing a Wilcoxon test for paired samples. Medians (—) and ranges (bars) are represented.

The T cell proliferation in presence of native and mutant forms produced a Th2 environment and the same T cell phenotype. In response to the four proteins, IL4, IFN*γ*, and IL10 were produced in the same way (*P* = 0.605, *P* = 0.362, and *P* = 0.107, for *Pru p 3.01*, *Pru p 3.02*, and *Pru p 3.03*, resp.) with more production of IL4 than IFN*γ* (Figure 5(a)). In this regard, the expression of the transcription factors for Th1, Th2, Treg and Th17, T-bet, GATA-3, Fox p 3, and ROR*γ*t, respectively, was measured by RT-qPCR (Figure 5(b)), and no significant differences were observed.

## 4. Discussion

Allergen-specific immunotherapy (SIT) represents the most effective treatment available toward allergies. However, in food allergy, SIT has not been practiced, mainly for the high risk to develop anaphylactic side effects for patients [13]. A few years ago, a pioneering study based on sublingual immunotherapy (SLIT) was performed in peach-allergic patients, using extracts with quantified amounts of Pru p 3 [14]. The obtained results were limited, probably because higher amount of allergens and a longer treatment would be required for more effective immunotherapy. However, the use of high doses of wild type allergens could present a serious risk for sensitized patients. This fact and the expensive cost of this type of treatment do not make their application in immunotherapy a very popular practice among clinicians.

By contrast, the use of hypoallergenic molecules may prevent these side effects and reduce cost, allowing us the use of higher doses of proteins and longer treatments. These features reveal them as a promising strategy to develop [6, 32]. Hypoallergenic forms could be obtained by different methods such as the search of native isoforms or molecular design modifying the wild type allergen [47, 48].

The LTPs syndrome is an allergy mediated by members of this family present in a wide range of plant foods and some pollen [25]. This means that people sensitized to LTPs should remove essential foods from their diet to avoid adverse reactions. However, the avoidance, sometimes, does not ensure unwanted contacts due to allergen traces in some processed foods. Nowadays, there are no effective treatments that improve the quality of life for these patients.

In this work, three Pru p 3 mutant forms have been produced by site-directed mutagenesis of the essential residues for IgE binding activity [29, 31]: *Pru p 3.01*, with four changes in the central region; *Pru p 3.02*, with four mutated residues in the C-terminal coil; and *Pru p 3.03*, including all modified residues of the other two. The three mutants retained their ability to bind blocking antibodies and to stimulate T cell proliferation. One of the mutants, *Pru p 3.01,* had a similar behaviour as the wild type, meaning that the four modified residues were not enough to abolish the allergenic capacity of the wild type form in a significant way. The other two, *Pru p 3.02* and *Pru p 3.03*, showed a significant reduction in their allergenicity capacity, since they had less ability to bind IgE and less recognition by patients with peach allergy, compared to the wild type protein. The reduction in cellular response by basophil activation test in these mutants suggested that their modifications were affecting the cross-linking on the surface of the effector cells.

In immunotherapy, several studies have indicated the importance to maintain the binding to IgG blocking antibodies. Those antagonize the recognition of wild type allergens by IgE antibodies captured on the surfaces of mast cells and basophils, which triggers the allergic reaction [33].

In the case of the mutants produced in this study, *Pru p 3.01, Pru p 3.02*, and *Pru p 3.03,* their ability to bind IgG1 was retained. In fact, some patients showed higher IgG1 binding to *Pru p 3.02* and *Pru p 3.03* than to wild type, probably due to the fact that some modified residues produced better recognition to IgG1.

In the case of IgG4, while *Pru p 3.01* and *Pru p 3.02* rendered no differences comparing to the wild type, *Pru p 3.03* showed a reduced ability to bind it. This behaviour may be explained in terms of overlapping between IgE and IgG4 epitopes, or because the loss of folding observed in this form could alter the binding to IgE and IgG4 but not to IgG1. In a recent paper [17], a reduced form of Pru p 3 was developed as a recombinant protein by modification of its six cysteines present in the primary structure. This unfolded mutant exhibited the ability to bind specific IgGs from sensitized mice (IgG1 and IgG2) completely abolished. Thus, the loss of 3D structure may imply the loss of some conformational epitopes, especially affecting IgE and IgG4 epitopes, in our *Pru p 3.03* mutant.

Besides the loss of IgE binding and the maintaining of blocking IgG recognition, it is essential for a successful immunotherapy that the hypoallergenic mutants also retain their capacity to stimulate the immune system [49], preserving their T cell epitopes. In our case, it was critical because one of the main T cell epitopes described in Pru p 3, Pru p 3_65–80_ [30, 50], was located in the same region as the C-terminal epitope. In the case of *Pru p 3.01*, the mutations did not affect T epitopes, and the T cell proliferation was not affected, as predictable. By contrast, the modifications in *Pru p 3.02* and *Pru p 3.03* could have interfered. However, both retained their ability to stimulate the proliferation of specific T cell lines, suggesting that their modified residues did not affect T cell epitopes.

Summarizing, in this work we have generated two hypoallergenic forms, *Pru p 3.02* and *Pru p 3.03*, which could be promising tools to be used in immunotherapy for peach-allergic patients.

*Pru p 3.03* had the highest hypoallergenic capacity of the three mutants. However, this mutant showed a loss of its three-dimensional structure, which made it more unstable in solution. This could hinder future applications. But also, this loss can be the cause of the drop in its IgE and IgG4 binding capacity, though preserving the IgG1 epitopes. By contrast, *Pru p 3.02* retained its ability to bind IgG antibodies, both IgG1 and IgG4, but exhibiting a considerable reduction of its allergenic activity, in both IgE binding capacity and patients' recognition. These characteristics make it highly recommendable for use in immunotherapy.

Although, for both *Pru p 3.02* and *Pru p 3.03*, more detailed studies such as their use in animal models are necessary to test their effectiveness, the approach presented in this work may be a first step towards the implementation of a new strategy for immunotherapy.

## Figures and Tables

**Table 1:** Demographic data and basophil activation to the native and mutant forms of Pru p 3 in patients with peach allergy.

Patient	Age (years)	Sex	% activated basophils (SI)*			
			1 *μ*g/mL			
			Pru p 3	*Pru p 3.01 *	*Pru p 3.02 *	*Pru p 3.03 *
1	40	F	18 (2.5)	—	—	—
2	38	F	24.3 (7.36)	—	—	—
3	55	M	20 (6.66)	21.8 (7.26)	20 (6.66)	22 (7.33)
4	39	M	24 (2.67)	23 (2.55)	—	—

F: female; M: male; —: negative response.

*% activated basophils ≥ 15% and SI ≥ 2 were considered positive response.

## Abbreviations

BAT: Basophil activation test
CD: Circular dichroism
ELISA: Enzyme-linked immunosorbent assay
LTP: Lipid transfer protein
PBMCs: Peripheral blood mononuclear cells
PBS: Phosphate buffer saline
SIT: Specific immunotherapy
TCLs: T cell lines.
